# Dataset for studying gender disparity in English literary texts

**DOI:** 10.1016/j.dib.2022.107905

**Published:** 2022-02-02

**Authors:** Akarsh Nagaraj, Mayank Kejriwal

**Affiliations:** University of Southern California, Information Sciences Institute, 4676 Admiralty Way, Suite 1001, Marina del Rey 90292 CA, United States

**Keywords:** Digital humanities, Text analytics, Natural language processing, Gender Disparity

## Abstract

Recent discourse has highlighted significant gender disparity in many aspects of economic, social and cultural life. With the advent of advanced tools in Artificial Intelligence (AI) and Natural Language Processing (NLP), there is an opportunity to use computational and digital tools to analyze corpora, such as copyright-expired literature in the pre-modern period (defined herein as books published approximately between 1800 and 1950) from the Project Gutenberg corpus. Nevertheless, there are challenges in using such tools, especially for maintaining high-enough quality to explore interesting hypotheses. We present a dataset and materials that illustrate how modern processes in NLP can be used on the raw text of more than 3,000 literary texts in Project Gutenberg to (i) extract characters and pronouns from the text with high quality, (ii) disambiguate characters so that they are not overcounted, (iii) detect the gender of each character. Furthermore, we also used manual labeling to determine the genders of authors who have published these texts, and published the labels as part of the dataset to facilitate future digital humanities research.

## Specifications Table

**Table:** 

Subject	Computer Science
Specific subject area	Computer Science Applications
Type of data	Text - Java Script Object Notation (JSON)
How the data were acquired	The raw data (text of out-of-copyright fictional texts) was acquired from Project Gutenberg (https://web.eecs.umich.edu/∼lahiri/gutenberg_dataset.html) [2], followed by systematic cleaning and preprocessing. Next, publicly available NLP (Natural Language Processing) libraries were used to segment sentences, extract characters and pronouns, and automatically assign gender to characters.
Data format	Analyzed Filtered
Description of data collection	The raw data is a subset of the Project Gutenberg books dataset [2], which is a digitized version of cultural works, processed and made available by researchers at University of Michigan. It consists of 3036 English books as text files, penned by 142 authors between 1700 and 1950.
Data source location	The primary data is available as a compressed .zip file containing 3036 text files, each pertaining to a single book in the Project Gutenberg dataset. This primary data can be downloaded from the following link: https://drive.google.com/file/d/1uPlm0JtJd9VipdUH7f-l1LjQ93VYRyAt/view. Since our work only relies on the .txt files that contain the text of the books, the data can also be directly downloaded from Project Gutenberg using the command ‘wget -w 2 -m http://www.gutenberg.org/robot/harvest?filetypes[]=txt&langs[]=en’ The books can also be *individually* downloaded from the page accessed at https://www.gutenberg.org/ebooks/search/?sort_order=downloads.
Data accessibility	Repository name: Project Gutenberg - Characters, Authors and their Gender [1] Data identification number: 10.17632/cmkmx2gzb3.1 Direct URL to data: https://data.mendeley.com/datasets/cmkmx2gzb3/1

## Value of the Data

• Our data enables gender-specific cultural analytics on pre-modern English literary texts obtained in raw form from Project Gutenberg. With a renewed focus on diversity and equity in the current era, understanding the lack of such diversity in cultural hallmarks, such as influential literary texts, is an important first step. Our dataset enables such analysis from the perspective of gender.
• The data will enable both digital humanities scholars and computational social scientists to quantify gender disparity in these texts both from a descriptive and statistical perspective. We expect that researchers currently studying gender disparity and gender bias in literature published in the post-Shakespearean era will benefit widely from this data.
• For each book that we analyze, we provide a list of characters with gender annotations. We also provide other details such as the (heuristically determined but highly accurate) gender of the author and distribution of gender-specific pronouns. This information will allow scholars to study gender disparity without having to master NLP tools or process large quantities of raw data themselves.

## 1 Data Description

The dataset is structured as a JSON text file containing metadata on 3036 fictional books as key-value dictionaries. Each such dictionary contains the title of the book as the main key, with the value being an ‘inner’ dictionary with the following information that have been obtained using the experimental design outlined in the next section:

**Table:** 

**Key (inner dictionary)**	**Description of corresponding value**

author_male	‘True’ if the author is male, and ‘False’ if the author if female
characters	List of all unique named characters in the book
character_count	{‘male’: number of unique male characters, ‘female’: number of unique female characters}
character_occurence_count	{‘male’: number of times male characters are referenced by name, ‘female’: number of times female characters are referenced by name}
pronoun_count	{‘male’: number of male pronouns in the book, ‘female’: number of female pronouns in the book}

A **supplementary data** file that is also included with the published repository [1] is a spreadsheet that contains the genders of the authors of the books described in the primary JSON file. The genders were determined manually by the authors, using both heuristics and public resources such as Wikipedia, since many of the authors have Wikipedia pages.

## 2 Experimental Design, Materials and Methods

Our main experimental objective in compiling this data was to extract and count all unique characters from a given corpus of modern English literary texts. A secondary aim was to detect the ‘gender’ of these characters, and count the numbers of times that they were mentioned in the book, as well as the counts of gender-specific pronouns in the books. Finally, we aimed to automatically detect the gender of the author of each book using a combination of semiautomatic heuristic techniques, including manual lookup using internet resources such as Wikipedia.

Because the corpus comprises more than 3000 books, automatic techniques are necessary for achieving these aims. We briefly describe these techniques, as executed in sequence, below. We also comment on the validation and expected accuracies of these methods.

For each book, as a first step, we split the input text into sentences to increase the performance of character extraction. To do so, we used a Python-based *sentence segmentation* module called *SegTok* that is capable of identifying sentence terminals such as ‘.’, ‘?’ and ‘!’, as well as disambiguating them when they appear in the middle of a sentence e.g., in the case of abbreviations and website links [3]. We evaluated the accuracy of SegTok by randomly sampling 110 sentence outputs that were segmented, and manually tagging them as being correctly segmented with respect to the paragraph in which the sentence was originally embedded. We found that, of these 110 sentences, only two were incorrectly segmented, leading to an accuracy of 98.18%.

Next, we extract named characters from each sentence using an NLP technique called Named Entity Recognition (NER). NER seeks to locate and classify named entities mentioned in unstructured text into pre-defined categories such as person names, organizations, locations, monetary values, to name a few. For this dataset, we are only interested in extracting person names from the text of the books. We do so by using the SpaCy package [4], in conjunction with the NE_Chunk package for tokenization [5].

Once extracted, we measure the number of times each character is mentioned in the book. In order to ensure we do not count the ‘same’ character (named in slightly different ways e.g., ‘Darcy’ and ‘Mr. Darcy’), we need to disambiguate the character extractions from the previous step. We used the *SequenceMatcher* class from the Python-based *difflib* library to do so [6]. This class specifically compares two strings and provides a similarity score between 0 (no match at all) to 1 (complete match, i.e., strings are the same). We treated string pairs, representing character extraction pairs, with a similarity score of 0.7 or above as duplicates. This threshold was selected after some sampling and manual verification. This disambiguation also allows us to count and record the number of *unique* characters extracted from each book. To assess its accuracy, we randomly sampled 76 character pairs that were disambiguated as duplicates by this heuristic technique, and found that 72 were correctly disambiguated, yielding an accuracy of 94.74%.

We also count the number of male (he, him, his) and female (she, her, hers) pronouns that appear in each book. Simple string-based pattern matching is used to accomplish this. We do not address gender-neutral pronouns in this dataset as they are largely absent from the books in the corpus due to the period, but they could be considered in future research that draws on more modern books to replicate the following analyses.

Finally, to classify the extracted characters as male and female, we used the Python-based *Gender_Detector* library developed using data from the Global Name Data project [7,8], which is able to determine the gender of a character from the first name. Using this library, we were able to heuristically tag each extracted character as male or female. We evaluated the accuracy of this method by randomly sampling 100 extracted characters (50 male and 50 female) and manually checking their actual gender against the predicted gender. There was only one error (of a male character), yielding an accuracy of 99%. However, this accuracy should only be treated as a preliminary estimate, since there are more than 50,000 characters across the full corpus of 3000+ books. We acknowledge this limitation further in the *Ethical statements* section.

Last but not least, all of the data obtained using the experimental design above is collected together into a JSON data structure [9], as discussed in the *Data description* section. The packages mentioned above are publicly available and can be easily used by anyone seeking to replicate our methodology on the same, or different, subset of texts obtained from digital preservation sources like Project Gutenberg.

## Ethics Statements

We acknowledge the possibility of bias in data processing and estimations of accuracy, which must be borne in mind by all those looking to use the dataset in future research. Specifically:

• One of the assumptions made by our data processing pipeline is that we can determine gender from the names of the book authors. This is obviously a simplification, and we caution researchers on relying upon this finding as their only source for determining the genders of book authors. Indeed, we hope that a more comprehensive study will point out the inaccuracies in our method. For this reason, we have made the full ‘raw’ data available in the repository.
• We also note that our accuracy estimates are derived from relatively small samples, out of thousands of data points. Hence, they may also be susceptible to certain biases. A study looking for higher power in its statistics should seek to compare against a larger sample, and we also advocate for acquiring manual annotations from multiple diverse individuals.
• Another important point is that we consider the simplistic dichotomy of male-female in determining gender, whereas it may be the case that there are non-binary and transgender authors and/or characters in the corpus. As better methods are developed for these classes, they must be used on the raw data to obtain more accurate classification results. This error may be amplified by not classifying gender-neutral pronouns, for instance.
• Finally, more investigation is needed on how our methods are skewed or biased in favor of one class (e.g., males, or books by male authors). In acknowledging these constraints and caveats, it is our hope and intent that future researchers will make ethical use of the corpus and the data in their research, rather than treat our accuracy and statistics as ground-truth, without further critical review.

## CRediT authorship contribution statement

**Akarsh Nagaraj:** Conceptualization, Methodology, Software, Validation, Data curation, Writing – original draft, Visualization, Investigation. **Mayank Kejriwal:** Conceptualization, Methodology, Supervision, Writing – review & editing.

## Declaration of Competing Interest

The authors declare that they have no known competing financial interests or personal relationships that could have appeared to influence the work reported in this paper.
